# “Homework Feedback Is…”: Elementary and Middle School Teachers’ Conceptions of Homework Feedback

**DOI:** 10.3389/fpsyg.2018.00032

**Published:** 2018-02-06

**Authors:** Jennifer Cunha, Pedro Rosário, José Carlos Núñez, Ana Rita Nunes, Tânia Moreira, Tânia Nunes

**Affiliations:** ^1^Departamento de Psicologia Aplicada, Escola de Psicologia, Universidade do Minho, Braga, Portugal; ^2^Departamento de Psicología, Universidad de Oviedo, Oviedo, Spain

**Keywords:** homework feedback, teachers’ conceptions, homework feedback purposes, perceived impact, focus group, classroom observations

## Abstract

This study explored mathematics teachers’ conceptions of the homework feedback focusing on four key aspects: definition, purpose, types, and perceived impact. Forty-seven teachers from elementary and middle schools participated in six focus groups. Data were analyzed using content analysis. To enhance the trustworthiness of findings, classroom observations were used for triangulation of data. Participants conceptualized homework feedback in three directions (i.e., teachers’ feedback provided to students, students’ feedback provided to teachers, and homework self-feedback), being teachers’ monitoring of students’ learning the purpose reported by most teachers. Participants also reported the types of homework feedback more frequently used in class (e.g., checking homework completion, checking homework on the board), and their perceived impact on students. Findings provide valuable information to deepen the understanding of the homework feedback process, which may help develop new avenues for future research.

## Introduction

Homework may be defined as tasks assigned by teachers to be completed in non-instructive time (Cooper, 2001), and has proved to enhance students’ academic achievement when endowed with particular characteristics (e.g., short, purposeful, frequent assignments, high quality) (e.g., Dettmers et al., 2010; Fernández-Alonso et al., 2015; Rosário et al., 2015a).

In addition, the homework feedback provided by the teacher in class is an important tool to increase the impact of homework on students’ learning and academic achievement (e.g., Walberg and Paik, 2000; Núñez et al., 2015b; Rosário et al., 2015b), and a crucial aspect of the quality of homework (Cooper, 2001). However, detailed information on elementary and middle school teachers’ perspectives about their practices and on the reasons why teachers choose and use particular types of homework feedback in class is still scarce (Bang et al., 2009; Deslandes, 2009; Rosário et al., 2015b). Investigating teachers’ conceptions of the homework feedback, particularly in elementary and middle school, may provide new insights into research on homework (e.g., helping further explain previous quantitative results; improving homework feedback measures), as well as into educational practices (e.g., teachers getting training on homework feedback practices).

### Teachers’ Role on the Homework Feedback Process

Teachers play an important role in the first phase of the homework process by setting up the objectives of homework assignments and designing tasks, and also in the final phase by implementing classroom follow-up practices (Cooper, 2001; Epstein and Van Voorhis, 2001; Rosário et al., 2015a,b). The latter includes, among other practices, homework feedback provided in class: oral or written praise, criticism, written comments (highlighting right and wrong answers), rewards, general review of homework in class, and grading (i.e., teachers giving numerical grades) (e.g., Elawar and Corno, 1985; Murphy et al., 1987; Corno, 2000; Cooper, 2001). These homework feedback practices are an important instructional tool for teachers in their teaching process (e.g., helping identify students’ difficulties, errors or misconceptions in homework; approaching the learning contents to accommodate students’ lack of prior knowledge, and redesigning homework to match students’ needs) (Corno, 2000; Walberg and Paik, 2000; Epstein and Van Voorhis, 2001; An and Wu, 2012; Bang, 2012).

Extant research lacks studies which have focused specifically on each of the above-mentioned types of homework feedback; still, some studies have shed some light on the usage and benefits of the various types of homework feedback. For example, Murphy et al. (1987, p. 68) found that “class discussion on homework,” and grading and commenting on homework were the practices most frequently used by high school teachers (i.e., English, mathematics, science, and social science) to monitor students’ completion of homework. Focusing on mathematics, Kaur (2011) explored the nature of homework tasks assigned by three 8th grade mathematics teachers (e.g., types of homework, sources of homework tasks), and found that teachers provided feedback on errors by grading assignments, orienting discussions and checking homework on the board when needed. Using the TIMSS 2003 data set, Zhu and Leung (2012) found that a high percentage of 8th grade mathematics teachers reported checking homework completion (85%), providing feedback regularly (i.e., at least “sometimes”, 100%), and discussing homework in class (96%). Nevertheless, none of these studies deeply explored the process of homework feedback.

### Students’ Role on the Homework Feedback Process

Students engaging in school tasks with autonomy and responsibility are expected to develop a sense of personal agency for self-managing their behaviors (Zimmerman, 1989). Besides, students who proactively manage their behaviors to attain self-set goals are likely to self-regulate their learning efficiently (Zimmerman, 1989). From a social cognitive perspective, self-regulated learning (SRL) may be defined as an active learning process whereby students self-set goals that direct their cognitions, motivations and behaviors toward those goals (Zimmerman, 1989; Núñez et al., 2013; Rosário et al., 2013). For example, robust self-efficacy and autonomy, good study skills, commitment to self-set goals, and positive academic attitudes are examples of core elements of academic self-regulation which are necessary to complete homework (Zimmerman and Kitsantas, 2005; Ramdass and Zimmerman, 2011; Schmitz and Perels, 2011; Núñez et al., 2015c). Regarding the latter, extant literature highlighted self-regulation competencies as essential tools not only to help students complete their homework, but also use the homework feedback delivered with efficacy and responsibility (Ramdass and Zimmerman, 2011; Zhu and Leung, 2012; Xu and Wu, 2013). In fact, students are given homework feedback in class and play an important role deciding what to do next with the information given (e.g., ignoring feedback, self-evaluating their homework performance, using SRL learning strategies).

However, to authors’ knowledge, research has not yet provided information to contribute to understanding how teachers’ homework feedback may promote students’ active role in the homework feedback process. As Corno (2000) reported, teachers are expected to promote students’ capacity to self-evaluate their homework, which would involve addressing important self-regulatory processes. Otherwise, homework feedback may fail to benefit students (Zhu and Leung, 2012).

### The Benefits of Homework Feedback

Research has analyzed the effect of specific types of homework feedback provided by teachers on students’ academic performance in a particular subject (e.g., Elawar and Corno, 1985; Rosário et al., 2015b), and also the relationships between homework variables (e.g., homework feedback perceived by students, students’ interest, homework management) using non-subject-centered designs (Xu, 2012; Núñez et al., 2015b). Focusing on the former (i.e., investigating homework feedback in a particular subject), Cardelle and Corno (1981) examined the effect of three types of written homework feedback (i.e., praise, constructive criticism, and constructive criticism plus praise) on college students’ written performance in a second language. Findings showed that students under the constructive criticism plus praise condition achieved a better written performance than their counterparts. Moreover, irrespective of performance levels (i.e., high, middle, and low), participants reported their preference for the constructive criticism plus praise condition. Elawar and Corno (1985) conducted a similar study in mathematics with 6th grade students. Findings showed that students under the constructive criticism plus praise condition showed better achievement and a more positive attitude toward mathematics (e.g., enthusiasm for mathematics) than students of the control group.

The synthesis by Walberg et al. (1985), and also recent findings by Rosário et al. (2015b), indicated that specific and individual feedback (i.e., giving written comments or grading homework) positively impacts students’ academic achievement. However, checking homework, grading, and providing individual feedback on homework assignments for every single student in class may not always be feasible because of teacher’s heavy workload (e.g., large numbers of students per class, large numbers of classes to teach, many school meetings per week) (Cooper, 2001; Rosário et al., 2015b). This educational constraint may help explain why checking homework on the board and checking homework orally are among the homework follow-up practices most frequently used by English as a Foreign Language (EFL) teachers (Rosário et al., 2015b). These practices are useful to teachers because they allow providing feedback to the whole class (e.g., Brookhart, 2008) with less effort than that needed to grade homework or comment on students’ assignments.

Moreover, homework feedback perceived by students was also investigated using non-subject-centered designs (e.g., Xu, 2011; Núñez et al., 2015b; for exceptions see Tas et al., 2016; Xu et al., 2017). In general, findings showed some of the benefits of homework feedback for students. For example, Xu’s studies using multilevel designs found that at student level teachers’ homework feedback reported by 8th and 11th grade students was positively associated with students’ interest in homework (Xu, 2008), students’ reasons for doing homework (Xu, 2010), students’ homework management (Xu and Wu, 2013; Xu et al., 2017), and students’ homework motivation management (Xu, 2014).

Analyzing students’ homework completion at 8th and 11th grade levels, Xu (2011) found a positive association with teacher homework feedback at both student level and class level. The explained variance was higher at class level. The author concluded that students’ homework completion is related to teachers’ provision of homework feedback (Xu, 2011). This proposition is further substantiated by the findings by Bang (2011), showing that high school immigrant students perceived teachers’ feedback as a facilitating factor, and the lack of it as an obstacle to homework completion.

More recently, Núñez et al. (2015b) conducted a study with students from various school years (grades 5–12) and concluded that the stronger the teachers’ homework feedback is perceived by students, the greater the amount of homework completed and the better the quality of homework time management (e.g., how well students managed time devoted to homework and avoided distractions). Moreover, these authors found that students’ academic achievement is indirectly and positively associated with homework feedback through students’ homework behaviors (i.e., amount of homework completed) and self-regulation (i.e., quality of homework time management), highlighting the importance of student engagement in the homework process (Núñez et al., 2015b). The results of Tas et al. (2016) are consistent with those, showing that middle school students’ homework self-regulation (e.g., orientation goals, learning strategies) mediated the relationship between perceived homework feedback and science achievement.

Bang (2012) reported that teachers acknowledged homework feedback (i.e., grading homework) as an important tool to motivate immigrant students to complete homework. Still, teachers admitted the educational challenge of providing homework feedback because of the time-consuming nature of this strategy. In fact, Rosário et al. (2015b) also reported the difficulties faced by EFL teachers to collect and grade homework on a regular basis. Both studies (Bang, 2012; Rosário et al., 2015b) called for further research on teachers’ perspectives about homework feedback.

In spite of the benefits of homework feedback for students previously reported, the literature has shown that teachers’ support in homework perceived by students decreases from elementary to middle school (Katz et al., 2010; Núñez et al., 2015b), without specifying in what aspects. Moreover, Kukliansky et al. (2016) recently observed middle school teachers’ behaviors in science classes (3–5 consecutive times) and found that in-class instructional feedback was not always provided, even when demanded by students. However, the authors did not explore the reasons why teachers did not provide feedback in this situation.

In sum, extant research on homework feedback has been conducted on controlled domain-centered contexts (e.g., Elawar and Corno, 1985; Rosário et al., 2015b), on single grade levels (e.g., Kaur, 2011; Zhu and Leung, 2012; Xu et al., 2017), is non-subject-centered (e.g., Xu, 2011), or explored specific populations (e.g., teachers of immigrant students, Bang, 2012) (cf. **Table 1**), thus further research is needed to deepen the understanding of the homework feedback process.

**Table 1:** Summary of studies that focus homework feedback.

Authors, date	Type of study	Participants	Domain(s)
Bang, 2011	Survey and qualitative study	High school immigrant students (9th–12th grade)	Non-subject-centered
Bang, 2012	Qualitative study	Teachers of high school immigrant students (9th–12th grade)	Several subjects (i.e., English, Mathematics, Science, Global Studies)
Cardelle and Corno, 1981	Experimental study	College students	Second language
Elawar and Corno, 1985	Experimental study	6th graders	Mathematics
Kaur, 2011	Qualitative study	8th grade teachers	Mathematics
Kukliansky et al., 2016	Qualitative study	Middle school teachers	Science
Murphy et al., 1987	Survey study	High school teachers	Several subjects (e.g., English, Foreign Language, Business Education, Mathematics, Science, Social Studies)
Núñez et al., 2015b	Correlational study	5th to 12th graders	Non-subject-centered
Rosário et al., 2015b	Quasi-experimental study	6th graders	EFL
Tas et al., 2016	Correlational study	6th to 8th graders	Science
Xu, 2008	Correlational study	8th and 11th graders	Non-subject-centered
Xu, 2010	Correlational study	8th and 11th graders	Non-subject-centered
Xu, 2011	Correlational study	8th and 11th graders	Non-subject-centered
Xu, 2012	Correlational study	8th and 11th graders	Non-subject-centered
Xu and Wu, 2013	Correlational study	8th and 11th graders	Non-subject-centered
Xu, 2014	Correlational study	8th and 11th graders	Non-subject-centered
Xu et al., 2017	Correlational study	8th graders	Mathematics
Zhu and Leung, 2012	Correlational study	8th grade teachers	Mathematics


*EFL, English as a Foreign Language.*

### The Present Study

Teachers are an important source of information in the study of the homework feedback process because they actually manage feedback in class (Cooper, 2001). Still, little is known about how mathematics teachers of different school levels perceive homework feedback. Examining elementary and middle school teachers’ conceptions of the homework feedback is expected to reveal useful information on the homework process, especially teachers’ beliefs and practices concerning homework feedback (cf. Irving et al., 2011). The model of teachers’ conceptions of assessment and feedback (Irving et al., 2011; see also Peterson and Irving, 2008) provides a relevant theoretical framework for the current research, and guided the research questions, data collection and analysis. This model addresses four key aspects of assessment and feedback: definition, purpose, personal response (i.e., types of assessment and feedback used) and perceived impact. Analyzing these key aspects focused on the homework feedback may provide data to help explain previous findings showing small effect sizes or low explained variances (see Zhu and Leung, 2012; Xu, 2014; Núñez et al., 2015b; Rosário et al., 2015b), and design future studies, homework policies or school-based interventions.

The following research questions guided the current study:

What are elementary and middle school teachers’ conceptions of homework feedback?

How do the four key aspects of the homework feedback relate to each other?

The current study explores the conceptions of teachers of two school levels for two reasons. Firstly, there are some differences as to the educational goals of those school levels; while teachers at elementary school focus on working on the foundations of mathematics (e.g., giving support in the development of number sense), middle school students are expected to learn high-level concepts (e.g., application of proportional relationships). Secondly, homework research found that the characteristics of the homework assigned (e.g., amount of homework assigned, homework purposes) vary for elementary and middle school. For example, Mullis et al. (2004) found that middle school students are expected to do larger amounts of homework than elementary school students. Besides, the purposes of assignments may also vary for both school grades. While homework purposes for middle school may be more related to school contents assessed in tests, homework purposes for elementary school are more likely to aim at developing personal skills such as time management (e.g., Muhlenbruck et al., 1999; Cooper and Valentine, 2001). Notwithstanding, the recent meta-analysis focused on mathematics and science by Fan et al. (2017) included a study in which elementary school teachers reported to assign homework to practice basic mathematics skills (see Bedford, 2014). Those differences (e.g., amount of homework assigned, homework purposes) may help explain the differential results regarding the benefits of homework in elementary and middle school (e.g., Cooper et al., 2006; Fan et al., 2017). Hence, elementary and middle school teachers were invited to talk about homework feedback in order to learn their conceptions and reported practices.

The current study focuses on mathematics (see Trautwein et al., 2006 on the importance of focusing homework research on a specific domain). The reason is threefold: students’ achievement levels, educational relevance of the subject, and previous research findings on homework. There is a global educational concern about students’ poor performance in mathematics. The PISA 2012 report indicates that students from 35 countries show a mathematics performance below the OECD average (OECD, 2014). This worrying educational scenario raises serious challenges for some countries (among which is Portugal), given the fundamental role played by mathematics in other subjects (e.g., biology, physics) and in the development of life and citizenship skills (e.g., Reyna and Brainerd, 2007; OECD, 2014; Hagger et al., 2015). Moreover, mathematics was chosen because of the great amount of homework that is regularly set by teachers (e.g., Trautwein et al., 2006; Schmitz and Perels, 2011; Xu, 2015).

## Materials and Methods

### School and Participants Characteristics

The last 2 years of elementary school in the Portuguese educational system encompass 5th and 6th grades (10 and 11 years old), while middle school includes 7th, 8th, and 9th grade (12–14 years old). Students have 270 min of mathematics per week in 5th and 6th grade, and 225 min per week in each of the three middle school years. At the end of 6th and 9th grade students complete a final exam that counts toward 30% of the overall grade.

Homework is an educational tool often used by Portuguese teachers as part of their lessons; still, there are no formal homework policies for Portuguese public schools (e.g., characteristics of homework assignments, homework follow-up practices; Rosário et al., 2015b).

In the current study, participants were involved in focus group discussions and some of them in classroom observations.

#### Participants in Focus Groups

Six focus group discussions were conducted in this study, each of which comprised 7–9 mathematics teachers. Three focus groups were set up with elementary school teachers (5th and 6th grade) and three focus groups with middle school teachers (7th, 8th, and 9th grade). Following Morgan (1997), homogeneity of groups was ensured in order to encourage participation among participants and minimize inhibition. Participating teachers met the following criteria: (i) having experience in teaching mathematics at elementary or middle school for at least 2 years, and (ii) assigning homework and providing homework feedback regularly (at least once a week). These requirements aimed to guarantee participants’ ability to generate ideas and opinions to share in their focus group.

The school administrators from the pool of schools which had previously enrolled in other university research projects were contacted by the authors. From those schools who agreed to participate, 20 public schools (approximately 25%) were randomly selected, and 75 mathematics teachers (approximately 25% of the pool of available elementary and middle school teachers) were randomly selected. Teachers were e-mailed about the purposes and procedures of the study (e.g., duration of the session, videotaping of the session) and invited to participate. To encourage participation (see Krueger and Casey, 2010), teachers were offered a participation reward (i.e., gift card), free baby-sitting services and a 3-h seminar on homework process and SRL to be held after the study had concluded.

In the end, 47 mathematics teachers (an acceptance rate of 63%) from 12 schools agreed to participate in the present study. The first author phoned the volunteer teachers to schedule the focus group meeting. Then, teachers were distributed into the various groups considering criteria such as: school, school level, and preferred scheduled time. Teachers with a hierarchical relationship were not allocated in the same focus group because this might affect their responses and the dynamics of the discussion (Kitzinger, 1995; Irving et al., 2011). In order to encourage attendance, all participants were reminded of the focus group session 1 week before and were asked to arrive 10 min early. A map with the location was sent to all participants.

All teachers attended the focus group discussions on the scheduled day (see **Table 2** for focus group demographics). Twenty-four teachers (51.1%) were teaching at elementary school level, and 23 (48.9%) at middle school level. In general, participating teachers had 21 years of teaching experience (*SD* = 6.11); taught students from middle-class families, as evidenced by the low percentage of students receiving free or reduced-price lunch (19.7%, data collected from the secretary’s office of the participating schools).

**Table 2:** Summary of demographic information of the focus groups.

Focus group (*N*)^1^	School level^2^	Gender^3^	Degree level^4^	Teaching experience	Number of classes	Workload^5^	Employment status^6^
1 (9)	M	3 M; 6 F	6 UG; 3 PG	14–30	3–5	5–27	Regular
2 (7)	E	1 M; 6 F	4 UG; 2 PG	23–38	2–3	14–21	Regular
3 (7)	M	7 F	5 UG; 2 PG	18–22	4–5	20–32	5 Regular; 2 PC
4 (9)	E	1 M; 8 F	9 UG	14–29	2–3	6–22	Regular
5 (8)	E	3 M; 5 F	6 UG; 2 PG	13–23	2–4	10–22	Regular
6 (7)	M	2 M; 5 F	6 UG; 1 PG	13–38	2–5	21–22	Regular


*^*1*^Number of Participants; ^*2*^School level: E – elementary school, M – middle school; ^*3*^Gender: M – male, F – female; ^*4*^Degree level: UG – undergraduate, PG – postgraduate; ^*5*^Hours per week; ^*6*^Employmeny status: PC – probationary contract.*

#### Participants in Classroom Observations

Given the time-consuming nature of observational studies, of these 47 teachers, 25% of the participants were randomly selected and asked to be observed in their mathematics classes. Finally, six teachers of each school level (*N* = 12; four males) were observed in their classrooms. These teachers had been teaching between two to five classes and they had an average of 19 years of teaching experience (*SD* = 6.93).

### Data Collection

Data was collected from two data sources: focus groups and classroom observations. The research team had previously enrolled in a qualitative research course offered by the University of Minho. Following a hands-on approach, the course training addressed topics including the following: how to lead focus group discussions (e.g., encouraging participation) and observations, and how to ensure the quality and credibility of a qualitative study.

This study was carried out in accordance with the recommendations of the ethics committee of the University of Minho. All subjects gave written informed consent to the different phases of the research (i.e., focus groups and classroom observations) in accordance with the Declaration of Helsinki.

#### Focus Group Discussions

Focus group interviews allow for in-depth exploration of meanings, attitudes, and personal experiences of participants about a particular topic during an informal, but structured, group discussion (Kitzinger, 1995; Krueger and Casey, 2010). This method of data collection helps capture teachers’ tacit knowledge in order to fill research gaps (Ryan et al., 2014). The focus group interviews were conducted by two members of the research team as facilitators while a third member filmed the sessions. To meet teachers’ availability requirements to participate, four focus group discussions were held at the end of the school year (July), and two at the beginning of the following school year (October). Each focus group session lasted approximately 60 min and took place in a room with appropriate light and sound conditions. To create a friendly environment, snacks and refreshments were offered to participants before and after the discussion. The chairs were arranged in a half circle to allow participants to see each other and to facilitate the filming of everyone in the room.

Prior to the discussions, teachers filled in a socio-demographic questionnaire (e.g., gender, years of teaching experience) and signed the written informed consent form. Then, the facilitators introduced themselves, read aloud the study purpose and the basic rules of the focus group discussion, and ensured confidentiality of participants’ responses (i.e., any information that may identify participants or their schools was eliminated at the end of the study).

To facilitate the interaction between participants, all focus group sessions started with a warm-up activity. Then, the facilitators started the discussion with general questions (e.g., the importance of homework) and, following Peterson and Irving (2008) and Irving et al. (2011), specific questions related to the four key aspects of homework feedback were asked: definition, purpose, types of homework feedback, and perceived impact (see **Table 3**). This set of questions was previously asked to two teachers in order to ensure comprehensibility. These teachers did not participate in the focus group discussions.

**Table 3:** Key areas and guiding questions used in teachers’ focus groups.

Definition	Purpose	Types of homework feedback	Perceived impact
● If you were asked to explain what homework feedback is, how would you describe it?	● In your opinion, what is (are) the purpose(s) of giving homework feedback?	● What type(s) of homework feedback do you usually provide?	● What do you think are the expected effects of homework feedback?
		What reasons lead you to give this kind of homework feedback?	What type of homework feedback do you think has more and less impact on students’ behaviors?
		● When do you usually give homework feedback to your students? And for how long?	● How do your students react to homework feedback?


*At the end of the questions, the facilitators asked participants if they wished to add anything to what had been said in the discussion.*

#### Classroom Observations

Classroom observations were conducted to capture teachers’ spontaneous behaviors regarding the homework feedback process. All invited teachers were informed that they would be observed five times on average (see Kukliansky et al., 2016), in a period of 3 weeks in the middle of the school year (March). Teachers were blind to the exact date or timetable of the observations (dates of the mathematics assessment tests were excluded from the observations schedule) and all agreed to participate acknowledging these requirements. Two other members of the research team, who were knowledgeable about homework research, conducted the classroom observations. These observations incorporated a structured content based on previous homework research to direct researchers’ attention to teacher’s responses to students’ homework completion (see Choo et al., 2015). The instrument used to collect data included the five homework feedback types reported in the literature (e.g., Rosário et al., 2015b). Additionally, researchers took field notes independently on the homework feedback process (e.g., time spent and how homework feedback was delivered), cross-checked and expanded upon their notes as promptly as possible. In the end, each teacher was observed on average five times, thus gathering a total of 64 h of classroom observations.

### Data Analysis

Transcriptions of focus group discussions and observation field notes were analyzed using content analysis (Bardin, 1996). The latter is a qualitative research technique used to search for and identify categories, following systematic procedures (Bardin, 1996).

The researchers who conducted the focus groups carried out the data analysis. Content analysis followed three main steps (Bardin, 1996): (i) reading the focus groups’ verbatim transcriptions to get an overview of the data (pre-analysis), (ii) coding (exploration of data), and (iii) treatment (e.g., percentages) and interpretation of data (e.g., comparing frequencies of coded categories). The organization, management, coding, and querying process of the data were conducted using the QSR International’s NVivo 10 software (e.g., Richards, 2005).

The extensiveness of comments (i.e., number of participants who convey an idea, Krueger and Casey, 2000) in the current study was the criterion used to identify categories. The identification of categories followed a deductive and inductive iterative process (Bardin, 1996). The categories were organized *a priori* in a coding scheme based on the theoretical model by Irving et al. (2011), and on the homework research (e.g., Walberg et al., 1985; Cooper, 2001; Xu, 2011; Rosário et al., 2015b). For example, the categories “definition,” “purposes,” “types,” and “perceived impact” of homework feedback were driven by the Irving et al. (2011) theoretical model, while each type of homework feedback (e.g., subcategory “checking homework on the board”) was driven by homework research (e.g., Rosário et al., 2015b). New categories were added during the analysis using participants’ words (Bardin, 1996). For example, the subcategories “homework feedback provided to teacher,” “self-esteem,” “homework self-feedback” were subcategories build upon teachers’ words. In the end, all transcripts were reviewed in order to check whether the already coded material fit the new subcategories.

Finally, the two researchers reviewed all the categories and sub-categories and discussed the differences found in order to reach a consensus (e.g., elimination of the subcategory “teachers assess students’ progress” because it was highly related to the subcategory “teachers monitor students’ learning”). After the data analysis of four focus group discussions (two from each school level), the researchers coded the two other focus group discussions and no new information was added. To ensure the reliability of findings, the Kappa value was calculated using the Coding Comparison Queries in the Navigation View of the NVivo software. The Kappa value was 0.86, which may be considered “almost perfect” according to Landis and Koch (1977, p. 165). Then data from the elementary and middle school teachers were analyzed separately conducting a matrix-coding query, crossing nodes with attributes (i.e., school level). The number of participants for each subcategory was converted into a percentage.

The two researchers who conducted the classroom observations coded independently the process of homework feedback delivery described in the field notes according to the codebook used in the focus groups. No new categories or subcategories were identified or redefined. Data from the elementary and middle school teachers were analyzed separately following the procedure used in focus groups, and the number of participants for each subcategory was converted into a percentage. To avoid bias on the Kappa value in NVivo, due to different numbers of characters of the researchers’ field notes (Kim et al., 2016), data was exported and IBM SPSS was used to calculate Cohen’s Kappa for nominal variables. The Cohen’s Kappa value for each subcategory ranged between 0.81 and 1.0, which indicates high agreement across observers.

To answer the second research question (i.e., How do the four key aspects of the homework feedback relate to each other?), data analysis followed two steps using the same software. First, a Cluster Analysis Wizard by word similarity between nodes was conducted to explore patterns and connections between nodes in an initial phase of data analysis (Bazeley and Jackson, 2013). Second, a case-by-nodes matrix was conducted to explore the relationships between each category in the focus group discussion transcripts as suggested by Bazeley and Jackson (2013).

Specific quality procedures were used to enhance the trustworthiness of the findings of the current study (Lincoln and Guba, 1985): investigator triangulation (i.e., several investigators were involved in the analysis process), methodological triangulation (i.e., patterns in data from focus groups and classroom observations were compared using a matrix-coding query, crossing nodes with classified sources – focus group and observations), and a member checking run at the University facilities. The researchers randomly selected and invited 25% of the participants of each grade level to do a member check (Lincoln and Guba, 1985). Ten teachers agreed to participate (six from elementary school and four from middle school) (response rate of 83%). Member checking session lasted approximately 2 h. Firstly, participants were informed of the findings (approximately 45 min). Afterward, they were given a copy of the findings and asked to analyze and discuss whether the description was an authentic representation of the topics covered during the focus group interviews. The participants also analyzed whether the description of the homework feedback types provided to students was an authentic representation of what usually happens in class. Participants were invited to critically analyze the findings and comment on them (Creswell, 2007).

## Results

**FIGURE 1:**
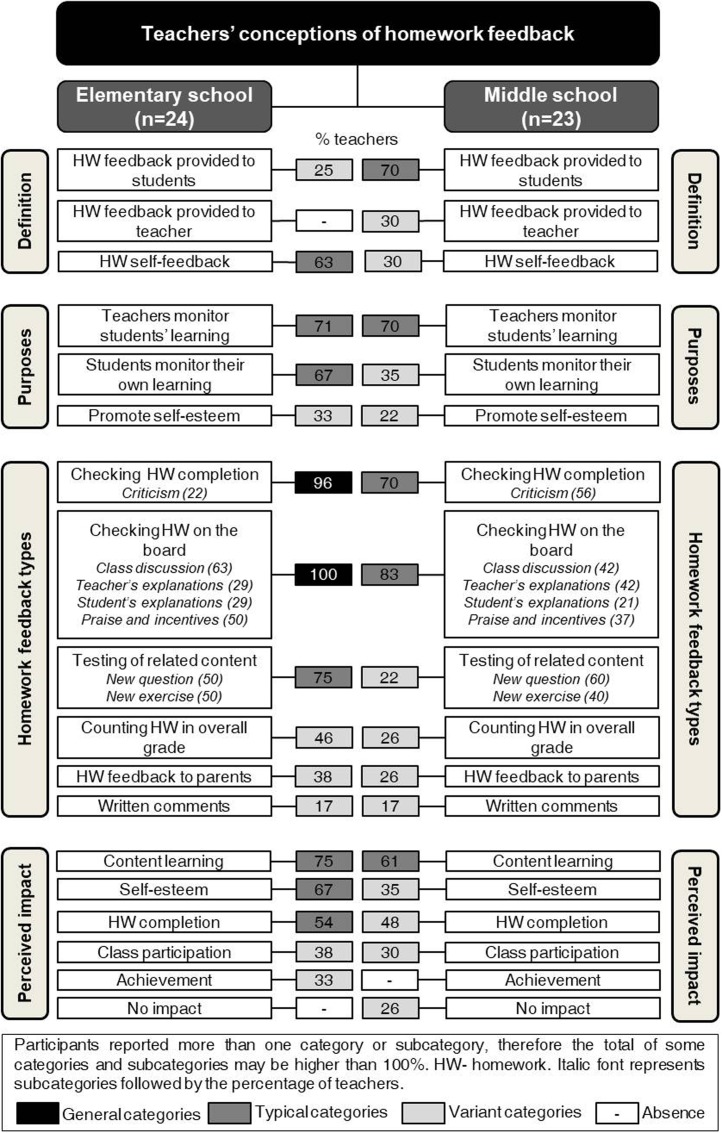
Elementary and middle school teachers’ conceptions of homework feedback for each key aspect.

**FIGURE 2:**
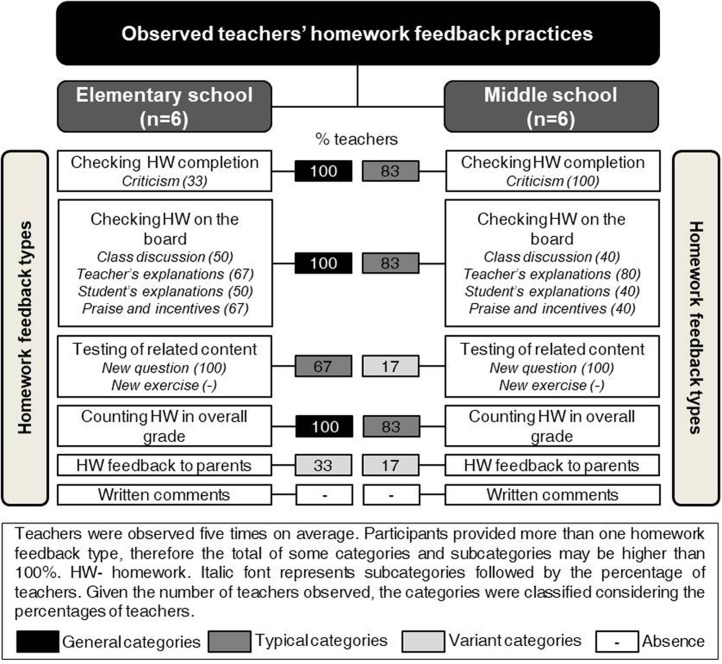
Observed elementary and middle school teachers’ homework feedback practices.

**FIGURE 3:**
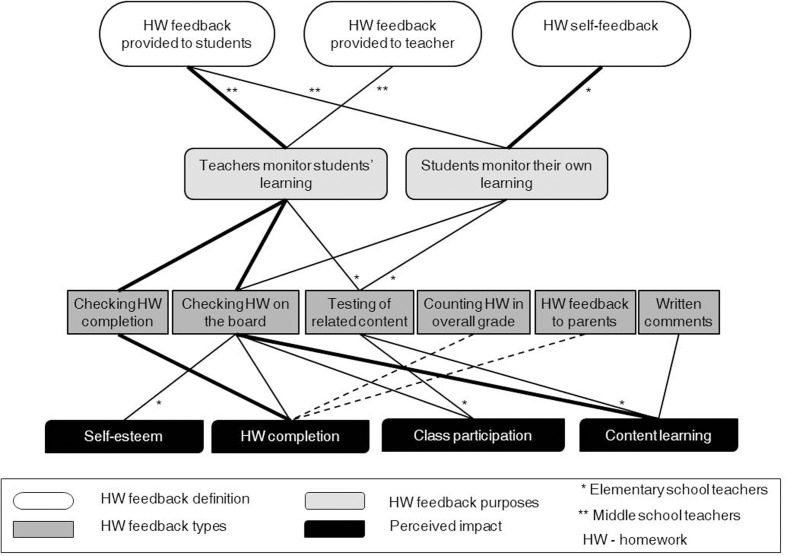
Relationships among teachers’ conceptions of homework feedback.

Data were organized and reported according to each of the key aspects of teachers’ conceptions of the homework feedback (see Peterson and Irving, 2008): definition, purpose, types of homework feedback practices, and perceived impact of homework feedback (see **Figures 1**, **2**). Furthermore, the relationships between these four aspects were presented (see **Figure 3**). Teachers’ verbatim quotes were introduced to illustrate the categories and conversations held in the focus group discussions (see also **Table 4**). In addition, whenever possible, data from classroom observations were included to illustrate findings. Categories were reported using the criteria by Hill et al. (2005, p. 16) as follows: general (i.e., categories include all, or all but one, of the cases), typical (i.e., categories include more than half of the cases) and variant (i.e., categories include more than three cases or up to half of the cases). For reasons of parsimony, rare categories (two or three cases) were not reported.

**Table 4:** Summary of findings.

Category	Subcategory	Description	Exemplar quotes from focus group
Definition	HW feedback provided to students	Information provided by the teacher to their students about homework behaviors, understanding and performance.	F1P4: Feedback is a concise message that teacher provides to students about their performance.
	HW feedback provided to teacher	Information provided by the students to their teacher about the process of homework completion and their performance.	F5P3: As I see it, feedback is the information that students give me regarding how homework worked with them…whether they had difficulties, whether they didn’t understand any statement of the exercise, the contents…
	HW self-feedback	Information generated by the students for themselves about their understanding of the content during homework completion or their performance during homework follow-up in class (e.g., when students compare their exercise solution with those written on the board, they can realize why is wrong).	F4P8: For me, homework feedback is internalized by students when they solve homework exercises correctly and understand whether they are (or not) on the right track.
Purposes	Teachers monitor students’ learning	Information regarding how teachers can learn students’ level of understanding of the contents taught in class; identify students’ misconceptions, their difficulties, and need of help (e.g., was the student able to do the exercise alone?) in order to respond (e.g., change teaching strategy) to students’ learning needs.	F3P7: Sometimes I go home and feel the lesson went very well; but when checking homework in the following day I may realize that only a few students understood the contents taught. That is when I try to explain the contents in a different way.
	Students monitor their own learning	Information regarding how students evaluate their understanding of the content by comparing their homework performance with the homework feedback provided (regardless the source – self or teacher).	F3P4: Sometimes they [students] think that their homework assignments are correct simply because they did them all, but often they are wrong … We have to call their attention… They have to check whether they really understood the material explained in class. Homework is like a written test at home…
	Promote self-esteem	Information regarding teachers’ efforts to promote students’ self-esteem (e.g., positive judgements about competence and positive feelings [pride]).	F5P4: I try to provide positive feedback to improve students’ self-esteem. When students have major learning difficulties, it is really important to note their progresses, even the slight ones, to help increase their confidence. Otherwise they may stop trying.
Types of homework feedback	*Checking HW completion*	Information regarding teachers’ efforts to check for HW completion. Teachers ask to all students of the class who did homework or give a quick check on students’ notebooks (e.g., walk around the students’ seats and glance their notebooks), and registered in homework logs who did not complete homework.	F1P2: I always begin the class, asking who did homework. I trust every student, but if I discover that someone didn’t complete homework, I will register a cross [mean “non-compliance”] in my homework log for all previous assignments.F1P9: I don’t do that because some students lie. I move around the class to control whether they really did homework.
	*Checking HW on the board*	Information regarding teachers’ efforts to check HW on the board. Teachers manage the checking of homework on the board (showing all steps), as follows: teachers solve homework on the board, teachers solve homework on the board following students’ instructions or teachers (randomly) ask students to (voluntarily or mandatorily) solve homework on the board.	F4P5: I always manage to check homework on the board. All students rotate on a regular basis to solve homework on the board. I ask them to detail all steps followed and I have a list to control students’ participation. The truth is… sometimes when I’m in a hurry I check myself homework on the board.
	*Testing of related content*	Information regarding teachers’ efforts to check students’ ability to transfer knowledge. When students present their homework completed in class, teachers ask questions about the contents focused on the homework assigned or provide new exercises to apply the content practiced in the homework assigned. Note: Any type of assessment is excluded.	F5P8: How many times did it occur? Students did their homework; homework was checked on the board, but eventually students did not really understand the contents… So I ask them to do a similar exercise, so students and I receive feedback regarding their understanding. Counterexample: F1P5:I only can really check students’ understanding on assessment tests.
	*Counting HW in the overall grade^∗^*	Teachers consider students’ homework completion or students’ performance when they check homework on the board to calculate students’ grade in the end of each term. Note: To collect this information, teachers use homework logs.	F4P4: Completing homework worth 5% to the final grade. The school grade regarding homework completion is the feedback that they receive.
	*HW feedback to parents*	Information regarding teachers’ efforts to provide HW feedback to parents. Teachers write a message on students’ notebooks to inform parents that their child did not complete homework.	F1P3: I also provide feedback to parents. When students do not complete homework I send a message to their parents communicating their child’s behavior.
	Written comments^∗∗^	Information regarding teachers’ efforts to provide HW feedback using written comments. Teachers write comments on students’ homework assignment claiming their attention to their mistakes, criticizing cheating, pointing positive aspects, and providing suggestions for improvement.	F4P4: When I teach geometry, I ask students to complete homework in a separate piece of paper. For example, in the assignment I may write “You did not use the protractor well. You have to pay attention to how to use it”.
Perceived impact	Content learning	Information regarding how teachers perceive the impact of HW feedback on students learning process and achievement. Teachers report to notice that homework feedback promotes students’ understanding of the contents, which enhances learning; homework compliance (i.e., completion rate and frequency), class participation (ask questions, participate in class discussions, answer teachers’ questions), self-esteem (i.e., positive judgements about competence and positive feelings), and achievement (i.e., grades on assessment tests, final grade).	F2P7: Effective feedback impacts on students at several aspects. I don’t have data to support it, but I can tell that students understand the contents taught and are likely to work harder than their mates to complete homework and participate in class…
	HW completion		
	Class participation		
	Self-esteem		F3P5: If we provide positive feedback, a smile appears… their self-esteem grows stronger little by little.
	Achievement		F5P7: (…) in the end students can get better grades in the assessment tests.
	No impact	Teachers refer that when students are not willing to learn, homework feedback may not impact on their learning process.	F6P1: There are students who do not really want to learn; for those students our feedback does not have any impact.


*HW, Homework. Italic font represents the homework feedback types observed during classroom observations. ^∗^Inferred through the usage of homework logs and teachers’ comments calling students’ attention that homework completion counts to their final grade. ^∗∗^Teachers that reported to comment on students’ homework regularly were not in the group of teachers who were randomly selected to be observed. The remaining teachers that reported this practice usually comment on the homework assignments focused on geometry, but classroom observations did not capture geometry lessons.*

### Initial Data Screening

All participants reported assigning homework regularly and considered homework feedback as an important element for homework effectiveness. Data showed that, for each homework assignment, 96% of the elementary school teachers and 52% of the middle school teachers reported spending approximately 30 min giving homework feedback in class. Moreover, 48% of the middle school teachers spent on average 15 min giving homework feedback in class. Classroom observations provided precise information on the time spent in class giving homework feedback: 3–80 min in elementary school classes (*M* = 32.75; *SD* = 19.91), and 5–55 min in middle school classes (*M* = 29.89; *SD* = 17.36).

### Definitions of Homework Feedback

When teachers were asked about their definition of homework feedback, the majority said they “had never thought about it” (F1P1). Still, elementary and middle school teachers elaborated on homework feedback differently (see **Figure 1**). Teachers from elementary school proposed two meanings for homework feedback: (i) homework feedback provided by the teacher and (ii) students’ homework self-feedback. For middle school teachers, homework feedback was conceptualized as threefold): (i) homework feedback provided by the teacher; (ii) homework feedback provided by the student; and (iii) students’ homework self-feedback. The analysis of the frequency labels for each category revealed no general categories, which allows concluding that definitions of homework feedback vary among teachers, irrespective of the grade level. Moreover, while “homework feedback provided to students” is a variant category in elementary school, in middle school is a typical category (see **Figure 1**).

For elementary school teachers in one focus group discussion and for middle school teachers in two focus groups, homework feedback provided by teachers was defined as a message provided to students with information concerning their homework behaviors (i.e., completion, effort), and comprehension of homework tasks and performance (e.g., how well students answered, why answers are wrong).

Middle school teachers in all focus groups conceptualized homework feedback in the reverse direction (i.e., from the students to the teacher), as the following statement illustrates:

F5P2: Some weeks ago, I noticed that several students in class had not understood some homework exercises. I asked the whole class and found out that no one had understood. Two or three students said: Sir, these exercises were a bit complicated… We did not understand what we were expected to do, how to start… This was the homework feedback they gave me.

The remaining teachers nodded their heads in agreement and added that this piece of information gathered at the beginning of a lesson helps them choose the type of homework feedback to give to students.

Lastly, elementary school teachers of two focus groups, and middle school teachers of one focus group (see **Figure 1**) proposed another meaning for homework feedback: “homework self-feedback” (typical category in elementary school and variant category in middle school). The following utterance illustrates this conceptualization:

F2P3: Homework feedback is when students can explain or reflect upon what they are doing…or checking from their seats when we check homework on the board.

Another elementary school teacher elaborated on students’ homework self-feedback:

F4P4: Homework feedback is also related to students’ homework completion. All my students draw a grid in their notebooks and devote one row to homework. Every day they write 1 for “completed” homework and 0 for “missing.” At the end of the term they have a score. I believe this to be self-feedback because students know their score and link it to school grades. They know that those who complete homework are likely to achieve better results. The opposite is also true….

This type of homework feedback (i.e., self-feedback) is more focused on students’ homework behaviors than on students’ homework performance. Still, other teachers from the same focus group reported that they do not use this strategy with their students.

### Purposes of Homework Feedback

The homework feedback purposes identified by teachers at both school levels were similar. Teachers enthusiastically talked about homework feedback as a “working tool” serving three main purposes (see **Figure 1**): (i) teachers monitor students’ learning and behavior (typical category in both school levels); (ii) students monitor their own learning (typical category in elementary school and variant category in middle school); and (iii) promotion of students’ self-esteem (variant category in both school levels).

When asked to expand on this idea, participants explained that homework feedback helps teachers identify students’ difficulties and monitor their content knowledge, which provides information to self-evaluate the instruction process and introduce changes if necessary. In fact, some students struggle to learn and show difficulties to understand and complete homework. To promote students’ motivation to do homework, teachers agreed on the need – “after charging our batteries of patience” (F6P3) – to explain in class how to do homework exercises. Besides, teachers exemplified the usefulness of homework feedback for monitoring students’ homework behaviors (e.g., checking whether students have completed their homework, whether they have copied the solutions from a textbook). This category emerged in all focus group discussions, and was consensual among participants. Teachers emphatically agreed on the examples discussed and expanded on others’ ideas. The following statements illustrate some of the conversations held:

F2P4: Homework feedback is important in order to learn about what is happening on earth [some teachers laughed], to learn whether most students do their homework, whether they manage to do it alone or need some help, but also to learn about their difficulties during the learning process and act upon their mistakes.
F2P1: To know whether I delivered the message well or not so… Homework feedback should make us change our instruction methodologies. If the message was not properly delivered, it’s necessary to change the course of action…

Moreover, many elementary and some middle school teachers in all focus groups mentioned students’ monitoring of their own learning as an important purpose of homework feedback, as illustrated by the following opinion:

F5P3: With the help of homework feedback, students can learn what is right or wrong in their homework. If the homework assignment is correct, they get some positive reinforcement. If it is not correct, they learn that they have to study more and do additional exercises.

Some points made by participants focused exclusively on one of the two previous purposes (see **Figure 1**). However, some teachers in all focus groups irrespective of grade level considered homework feedback a purposeful tool for teachers or students to monitor progress in learning. In sum, teachers admitted that homework feedback provides on-task opportunities for teachers and students to monitor the teaching and learning process.

Moreover, teachers from five focus groups pointed at the promotion of students’ self-esteem as another purpose of homework feedback (see **Figure 1**). Elementary and middle school teachers supported this idea, showing concern about students’ wellbeing, mainly of low achievers:

F5P6: When they [students] realize that they are capable of doing homework exercises, they feel very happy and proud of themselves. When they fail to complete or feel frustrated because they couldn’t find a way to do the exercises, I try to make positive comments, highlighting what they did well in order to make them feel confident. It is crucial to give them positive reinforcements to improve their self-esteem.

### Types of Homework Feedback Practices

Going further in the discussion, teachers identified the most frequently used homework feedback practices: (i) checking homework completion; (ii) checking homework on the board; (iii) testing related content; (iv) considering homework in the overall grade; (v) informing parents of their children’s homework non-compliance (homework feedback to parents); and (vi) giving written comments (see **Figure 1**).

The two types of homework feedback practices first mentioned in all focus group discussions were: checking who completes homework and checking homework on the board. The classroom observations (see **Figure 2**) provided information on the classroom routines and, with some exceptions, allowed concluding that classes usually begin with similar routines: checking who did homework and then checking homework on the board.

As **Figure 1** shows, checking homework completion is a general and typical category among elementary and middle school teachers, respectively. When discussing this practice (see **Table 4**), some of the elementary and middle school teachers argued that they simply ask who completed homework. On the other hand, most elementary and some middle school teachers explained that they walk around the class having a glance at students’ notebooks in order to check homework completion. This strategy allows noting who actually did their homework and gathers information on how students did it (e.g., whether students followed all the steps to solve a problem). In this process, teachers reported that they try to understand the reasons why students did not complete homework (e.g., is failing to complete homework a class problem or is it only associated with a particular student?). The participating teachers considered this type of homework feedback useful because it gives information on the process and allows them to respond to students’ maladaptive homework behaviors (e.g., missing homework, copying solutions from peer students, writing down results without checking). Teachers from both school levels reported using logs in class to record who missed homework, and data from the classroom observations corroborated this finding. When asked how they usually deal with maladaptive homework completion behaviors, some teachers at both school levels reported criticizing students who repeatedly fail to complete homework or copy answers from the textbook, as the following utterance illustrates:

F6P5: Where is your homework? Oh, I see. Keep working like this and you will get far… [ironic tone]

The use of public criticism and irony in response to maladaptive homework behaviors was observed sometimes in elementary school classes, and often in middle school classes.

When discussing the best practice regarding homework, participants at both school levels named checking homework on the board as a practice that “reaches all students” (F6P4). All teachers were very emphatic about the importance and usefulness of this type of feedback. As **Figure 1** shows, this practice is the most frequently used by elementary and middle school teachers. Moreover, present data (i.e., focus group discussions and classroom observations) suggest several ways in which this type of homework feedback may be put into practice. For example, some teachers reported that they check homework on the board; others mentioned writing on the board the answers dictated by students from their seats; while others explained that they randomly choose one or more students to do homework exercises on the board. Elementary and middle school teachers further explained additional homework feedback practices adopted after displaying the solution for the exercise on the board: (i) whole-class discussion led by the teacher; (ii) further explanation provided by the teacher or by the students on what is written on the board; (iii) teachers’ praise for students’ efforts in learning or good performance, or (iv) general incentives encouraging students to persist when doing homework. The observations conducted in the classrooms provided data that showed that all these strategies were used in class when teachers were checking homework on the board. Still, frequency and sequence of the strategies used by teachers (e.g., students check homework on the board, teacher explains problem solving procedures, class discussion) varied according to the needs and characteristics of the class. Moreover, classroom observations revealed that when students ask teachers for help, some teachers provide individual explanations while checking homework on the board. For example, when students raise their hand to show a lack of understanding while checking homework on the board, some teachers go to the student’s desk to answer their question individually.

Teachers at both school levels also emphasized checking homework on the board as a way of giving feedback to the whole class with minimum time and effort:

F1P1: When homework is being checked on the board by a student, I identify what is incorrect and explain how the exercise may be approached. Still, this feedback is very general because I cannot check every single assignment that students hands in. I simple cannot do it!

However, some participating teachers alerted that students who check homework on the board get a more detailed type of feedback than those who passively watch from their seats or do not pay attention to the checking process.

Moreover, many elementary and some middle school teachers in all focus group discussions mentioned asking questions, or assigning exercises similar to those of previous homework assignments (see **Figure 1**, Testing of related content). Data from the classroom observations confirmed this practice. Participants stressed that this practice provides students with a new feedback event centered on the level of accuracy of their responses and on their ability to transfer the knowledge learned to new tasks. However, despite the general agreement regarding this homework feedback practice, some middle school teachers admitted that they only check students’ ability to transfer knowledge in assessment tests and claimed that this practice should not be considered homework feedback – “This is assessment, not feedback! [Emphatic tone]” (F1P5).

Most participants at both school levels reported following their school’s assessment criteria regarding homework. Generally, homework completion counts for 2–5% of the overall grade in mathematics. When asked to be more specific, several teachers explained that they use information on homework completion recorded in class logs, while others declared using information on students’ performance when checking homework on the board. Teachers admitted that they do not examine the quality of all homework assignments given in class because of the heavy workload they faced on a daily basis. During classroom observations, teachers registered who did not complete homework and sometimes they referred that this behavior would decrease their overall grade. Most teachers in all focus groups reported including information on homework completion in the overall mathematics grade; still, less than half identified this practice as a type of homework feedback.

Furthermore, some of the elementary and middle school teachers in all focus groups mentioned sending parents a message when their children miss homework three times as a type of homework feedback. This practice was confirmed by data from classroom observations. Interestingly, participants did not mention reporting children’s progress on homework to parents during the focus group discussions, and accordingly this practice was not observed in class.

Finally, a few elementary school teachers in two focus group discussions and a few middle school teachers in one focus group reported commenting on students’ homework regularly. Comments address the strengths and weaknesses of homework, pointing out the topics that need to be improved, as the following quotation exemplifies:

F4P1: I comment on what is done well, but I also point out mistakes and suggest ways to improve what is wrong or not so well done. For example, I’d write: “Great line of reasoning but try to do x so you’ll only have to do two calculations and you’ll finish the exercises faster.” Unfortunately, sometimes I have to write other kinds of comments such as “What a coincidence, your answer is exactly the same as Joana’s or Catarina’s … and the three of you have made exactly the same mistakes…”

These few participants were asked by their focus group peers how they managed to comment on students’ homework regularly. A teacher answered that she could do it because she had been assigned only one class; still, “I spent my lunch hour and some of my free time at school working on this” (F3P3). Another teacher explained that she provides this type of homework feedback weekly, except for those weeks when students have assessment tests. According to this last participant, the negative side of this practice, “frustrating I should say” (F4P1), is when students copy homework answers from another student. Commenting on students’ homework is a very time-consuming practice, and this participant expressed feeling discouraged when such maladaptive behaviors occur in class. To overcome the “very time-consuming obstacle,” another teacher who also claimed to use this practice explained that he usually asks the whole class to complete homework on a separate sheet – “I choose only one good exercise which reflects the material covered in class” (F5P7). In the next lesson, and without prior notice, he collects four or five homework assignments, which are returned with feedback comments in the following class. Participants in the three focus group discussions agreed that this type of homework feedback is very useful, but also stressed the unlikelihood of giving it in class because of the heavy workload they as teachers have to bear (e.g., teachers have to teach five or six classes at different grade levels, each of them with over 25 students, heavy curriculums). In this context, one participant complained: “I’m not a rubber band that may be stretched [endlessly]” (F5P5).

### Perceived Impact of Homework Feedback

As **Figure 1** shows, elementary and middle school teachers highlighted the positive impact of homework feedback on content learning, self-esteem, and homework completion (some categories are typical and others are variant).

The following dialog among elementary school teachers illustrates their conceptions on the impact of homework feedback:

F4P9: Students who complete homework regularly are more willing to understand the contents covered.
F4P2: …and they complete homework more often… At least I notice more effort.

Moreover, both elementary and middle school teachers related homework feedback to class participation (variant category in both school levels), as the following participant argued:

F4P5: Yes…they [students who complete homework regularly] follow classroom instructions and participate in class more actively, for example, by asking me questions and answering mine more frequently…

Only elementary school teachers in two focus group discussions related homework feedback to students’ achievement, while none of the middle school teachers did so (see **Figure 1**). In fact, some of the middle school teachers in all the focus group discussions defended the need for students to play an active role in their learning, arguing that homework feedback is not worthwhile for those who are not interested in learning.

### Relationships between Teachers’ Conceptions of Homework Feedback

The second research question aimed to examine how the four key aspects of the homework feedback are related. **Figure 3** provides a graphical model of teachers’ most salient conceptions of homework feedback and the relationships among them. The bold solid lines represent typical cases (more than 50%), the thinner solid lines represent variant categories (between 25 and 50% cases), and the dotted lines represent variant categories (between 17 and 24% cases). All lines represent the conceptions of both elementary and middle school teachers except for the lines with an asterisk, which refer to a specific school level (see legend of **Figure 3**).

As **Figure 3** shows, the definitions of homework feedback provided by elementary and middle school teachers differ regarding the purposes for giving homework feedback. The middle school teachers perceived homework feedback as the feedback provided by the teacher to their students about their homework. The purpose for this homework feedback was described by teachers as twofold: help teachers monitor students’ learning and help students monitor their own learning. The latter was mentioned less often by middle school teachers. Besides, the middle school teachers conceptualized homework feedback provided to teachers by their students with the purpose of helping teachers monitor students’ learning.

In turn, elementary school teachers perceived homework feedback mainly as self-feedback and, accordingly, conceptualized students’ monitoring of their learning as the main purpose for giving homework feedback. While discussing, these teachers highlighted students’ active role in self-regulate their learning during and after homework completion (e.g., students checking their answers when solutions are written on the board). Still, the elementary school teachers did not explain how they promote these self-regulation skills in class. Moreover, the second set of relationships (i.e., purposes and homework feedback types) reveals a different pattern of results as described below.

Interestingly, the participating teachers operationalized both homework purposes (i.e., teachers monitoring students learning and students’ monitoring their own learning, the latter less often; see **Figure 3**) through the “checking homework on the board” homework feedback type. Teachers’ arguments were twofold: this practice allows checking students’ level of understanding of content (e.g., students solving exercises autonomously on the board), and students can learn about their skills while checking their answers with those written on the board.

The homework feedback practice testing related contents was also linked to both purposes but only by elementary school teachers. These teachers argued that providing students with similar exercises to those previously set as homework helps teachers monitor their students’ learning and students to monitor their own learning.

The purpose “teachers’ monitoring their students’ learning” was linked to the practice “checking homework completion” by elementary and middle school teachers. This homework feedback practice helps teachers learn who completed homework and collect information on the content with which students are struggling the most. This information is expected to help teachers meet their students’ needs.

Finally, the various types of homework feedback were associated with various perceived impacts. Teachers at both school levels converged in the fact that checking homework completion impacts students’ homework completion positively. In general, teachers mentioned that some students are “immature” and their lack of active involvement and strong volition prevent them from completing homework. Thus, most of the teachers at both school levels anticipated that external control is needed to help students complete homework. Checking homework completion was referred to as an important tool for encouraging students to do homework.

As **Figure 3** depicts, teachers described checking homework on the board as the homework feedback practice that most benefits students. According to participants, this practice fosters self-esteem (only reported by elementary school teachers), homework completion (reported by some teachers at both school levels), class participation (reported by some teachers at both levels), and learning of the content taught in class (reported by most of the teachers from both levels). Teachers explained that praising students on their good performance while doing exercises on the board is likely to increase their self-esteem. Furthermore, teachers said that this practice encourages homework completion and increases class participation because it provides students with specific information on how to solve exercises.

Some elementary school teachers reported that testing related content helps students participate more in class (e.g., answering teacher’s questions, asking more questions) and be more engaged in their learning.

A few teachers at both school levels (see **Figure 3**) mentioned counting homework in the overall grade, and communicating with parents when their children miss homework three times as two types of homework feedback with impact on students’ homework completion. Counting homework completion in the overall grade was referred to as a direct incentive for students to complete homework. However, some teachers alerted that this practice may not always be effective because of the time gap between students’ homework behaviors and the end of term when they get their final grade report. Thus, all agreed that teachers should respond to students’ homework behaviors (e.g., missing homework or doing assignments correctly) as soon as possible. Participants highlighted the importance of communicating with families about children’s homework behaviors. However, teachers alerted that this type of homework feedback may not be effective without the implication of the family in the learning process; “if the family is aware of the importance of this type of practice, then it will be effective, otherwise it will have no effect” (F3P4).

As reported previously, 17% of the elementary and middle school teachers claimed to make written comments on students’ homework assignments (see **Figure 1**). However, when discussing the possible impact of the various types of homework feedback, more teachers (than that 17%) agreed that written comments on students’ assignments would improve students’ learning of content (see **Figure 3**). These teachers mentioned that personalized homework feedback would help students correct their mistakes and might provide guidance on the topics that need to be further studied. As a result, students were likely to improve their grades.

## Discussion

The discussion of the current study is organized according to each key aspect of teachers’ conceptions of homework feedback. Regarding the first key aspect of homework feedback, teachers proposed a multifaceted definition of homework feedback: (i) homework feedback provided by the teacher, (ii) homework feedback provided by the student, and (iii) homework self-feedback. The latter extends the definition of Cooper (2001), who defined homework feedback as the teachers’ responses to students’ homework completion as a follow-up (e.g., comments, incentives, grades). The definition of homework self-feedback is linked to the internal feedback or self-feedback proposed by Butler and Winne (1995) and Hattie and Timperley (2007), respectively. According to these authors, students are expected to display self-regulatory skills to self-evaluate their performance in homework assignments (see Hattie and Timperley, 2007). Interestingly, this category is typical in elementary school, but variant in middle school. This is an important finding because the generation of internal feedback requires knowledge on strategies and standards, as well as the capacity to judge the quality of a task in relation to standards, which not all students are capable of, especially those at lower grade levels (Zimmerman and Martinez-Pons, 1990; Butler and Winne, 1995; Rosário et al., 2016). Moreover, low achievers struggling to learn often fail to activate and control the SRL process (Núñez et al., 2015a). In fact, these students are likely to fail to monitor their homework behaviors because they do not know “whether they are on the right track” (F4P8).

Consistently with literature, teachers’ major conceptions of homework feedback purposes addressed monitoring students’ learning, either focusing on teachers’ or on students’ role (Corno, 2000; An and Wu, 2012; Bang, 2012). This may be particularly important in mathematics where contents are organized so as to follow a continuous progression and lower levels prepare the foundations of subsequent levels (Pijls and Dekker, 2011). Teachers’ monitoring provides the opportunity for teachers to change their teaching practices in response to students’ needs (Walberg and Paik, 2000; Kralovec and Buell, 2001), which may be understood as a “student-centered” approach (see Sheridan, 2013). The conception of homework feedback purposes focused on students’ monitoring their work emphasizes students’ active role during the homework process and the use of SRL competencies such as self-monitoring and self-reflection (e.g., Ramdass and Zimmerman, 2011; Zhu and Leung, 2012). The last purpose of homework feedback proposed by participants is to “promote self-esteem.” This purpose is not mentioned in homework literature; however, in the study by Irving et al. (2011), teachers mentioned the need to inform students about the positive aspects of their performance, thus incentivizing their progress, especially among low achievers showing low self-esteem.

Regarding the third topic of homework feedback (homework feedback types), findings in the current study are consistent with literature (Cooper, 2001; Mullis et al., 2004; Kaur, 2011; Zhu and Leung, 2012; Rosário et al., 2015b; Kukliansky et al., 2016). However, despite the similarity of the homework feedback practices reported by elementary and middle school teachers, the percentages of each reported category vary. For example, checking homework completion and checking homework on the board are general categories in elementary school and typical categories in middle school; while testing of related content is a typical category in elementary school and a variant category in middle school. These findings are consistent with students’ reports on their teachers’ support in homework (Katz et al., 2010; Núñez et al., 2015b). A decrease in teachers’ support in homework at middle school level is expected because older students are likely to be more autonomous. However, Katz et al. (2010) found that the middle school students who perceived low teachers’ homework support reported high psychological needs and low intrinsic motivation. Other important finding to note is the use of criticism observed in elementary and middle school classrooms which may have the opposite effect of teachers’ intentions (e.g., reduce homework non-compliance). In fact, being criticized in class is likely to be non-constructive because it may reduce students’ willingness to accept criticism and result in low favorable responses toward homework. On the contrary, criticism delivered in private is likely to lead to better responses (see Leung et al., 2001).

According to participants, homework feedback impacts in the following aspects: content learning, self-esteem, homework completion, class participation, and achievement. Globally, this finding is consistent with previous research (e.g., Trautwein and Lüdtke, 2009; Xu, 2011; Núñez et al., 2015b), except for class participation and self-esteem which have not yet been studied. It is interesting to note, however, that despite most teachers reported spending 30 min or more providing homework feedback in each class (see Initial Data Screening subsection); about one third of elementary school teachers related homework feedback to students’ achievement, while none of the middle school teachers did so. However, prior research has evidenced the positive impact of homework feedback on students’ academic achievement (Núñez et al., 2015b), especially when teachers provide suggestions on how to improve learning (see Elawar and Corno, 1985; Walberg et al., 1985; Rosário et al., 2015b).

Moreover, middle school teachers added that when students do not play an active role in their learning, feedback is not likely to have any impact. This conception is consistent with the SRL approach to the homework process (e.g., Xu and Wu, 2013; Xu, 2014; Núñez et al., 2015b) which stresses, for example, the role that teachers may play in helping students define their own homework goals and reflect on the relationship between homework completion and achieving self-set learning goals (e.g., Núñez et al., 2015b; Rosário et al., 2015b). As Labuhn et al. (2010) observed, the feedback provided by teachers may not impact students’ learning and behaviors if students do not understand what is intended with homework feedback.

Findings gathered from relationships between teachers’ conceptions of homework feedback provide additional useful insights. Interestingly, the two most frequently reported types of homework feedback (i.e., checking homework completion and checking homework on the board) in both school levels are more linked to the purpose “teachers monitoring students’ learning” than to the purpose “students monitoring their own learning.” This data may suggest that teachers may not be fully aware of the importance of promoting students’ SRL competencies to increase the benefits of homework feedback or they may lack the knowledge to promote these skills in class (see Spruce and Bol, 2015).

### Practical Implications

The current study provides four major findings of relevance for educational practice: (i) decrease of teachers’ reported homework feedback practices from elementary to middle school level; (ii) a few teachers from elementary school and none from middle school level perceive homework feedback impacting on students’ academic achievement; (iii) usage of public criticism in class, especially in middle school; and (iv) teachers’ lack of awareness on SRL strategies.

First, teachers and school administrators with the help of school psychologists could examine homework practices delivered in class, namely homework feedback, to analyze whether they are set to be responsive to students’ educational needs. As found in the current study, there is a decrease of the homework feedback from elementary to middle school; however, this finding should be considered by teachers because, according to literature, many middle school students still report the need of teachers’ homework support (e.g., Katz et al., 2010).

Data also showed that both elementary and middle school teachers spend around 30 min providing homework feedback in class, but the perceived impact of this school practice on students’ achievement was barely mentioned in the focus groups. This data merit reflection within the school context to understand whether homework feedback is being used with efficacy. For example, school-based training for teachers’ on homework models (e.g., Cooper, 2001; Trautwein et al., 2006) could theoretically ground their homework practices in schools. This training would also help teachers understand that criticism and irony in class may discourage homework compliance, but it also may lead to undesirable outcomes such as children homework disengagement.

Finally, data (e.g., elementary school teachers believe that students generate homework self-feedback; the homework feedback practices most used in class are more closely related to the purpose “teachers monitoring students’ learning” than to the purpose “students monitoring their own learning”) suggest the need to set school-based training for teachers on SRL strategies. This training could consider addressing the homework process in relation with SRL to promote students’ agency and sense of responsibility over homework and homework feedback in particular. For example, teachers are expected to learn and practice how to model the use of SRL strategies in class (Rosário et al., 2013; Spruce and Bol, 2015). In fact, students lacking SRL skills may fail to use the homework feedback delivered in class, which may compromise the impact of this instructional tool on students learning and achievement (see Corno, 2000; Peterson and Irving, 2008; Zhu and Leung, 2012). To promote the development of students SRL competencies and increase the benefits of homework feedback, teachers may also consider using “diary tasks” to promote students’ homework self-reflection in class (see Ferreira et al., 2015).

### Strengths, Limitations, and Future Research

To authors’ knowledge, this study was the first to map mathematics teachers’ conceptions of homework feedback and examine the relationships between teachers’ definitions, purposes, types, and perceived impact of homework feedback. The analysis of these relationships focusing on a specific content domain at two school levels showed which categories were linked, and how, by the participating teachers. This study extended previous research conducted with mathematics teachers from a single grade level (see Kaur, 2011; Zhu and Leung, 2012).

According to the current findings, elementary and middle school teachers’ conceptions of homework feedback vary, as well as the time spent in class providing feedback. Moreover, in spite of the fact that the types of homework feedback practices are the same, the type of categories (i.e., general vs. typical and typical vs. variant) varies in the two school levels, and the dynamic of providing homework feedback at those school levels is diverse and complex (e.g., usage of various strategies to provide some types of homework feedback, even by the same teacher). These findings may help understand why the relationship between homework and academic achievement reported in the literature varies from elementary to middle school (see Cooper et al., 2006; Fan et al., 2017).

Furthermore, the complexity of the homework feedback process reflected by the collected data may not be captured by extant instruments that examine teachers’ homework feedback practices. To some extent, this may contribute to understand the low effect sizes and explained variances found in the homework feedback research (e.g., Xu, 2014; Rosário et al., 2015b). This finding reinforces the need for future studies collect data using more than one method to capture and better understand the phenomenon of the homework process and its influence on students’ academic outcomes (e.g., Cooper et al., 2006). Furthermore, findings showed positive relationships between some types of homework feedback practices and perceived impact on students’ variables that have not yet been examined in homework research (e.g., checking homework on the blackboard and class participation). Future studies may consider further examining these relationships.

The present study followed methodological procedures to enhance trustworthiness of findings such as random sampling, investigator and methodological triangulation, provision of direct quotations, and member checking (Lincoln and Guba, 1985; Shenton, 2004; Elo et al., 2014). Results from member checking were very positive. The majority of the participants agreed that the description of the findings was a genuine reflection of the topics covered in the focus group discussions, and of the homework routines in the classroom. No suggestions were made to change the description of data. Such data have strengthened present findings. In addition, teachers highlighted that they usually choose types of homework feedback that reach all students because of the professional constraints they experience daily (i.e., heavy workload). This topic was mentioned during the discussions and may merit further investigation because it may be an important factor compromising the homework feedback process.

Notwithstanding the strengths of the current study, there are also some limitations that need to be addressed. Classroom observations helped strengthen findings, nevertheless only 25% of the participating teachers were observed in a limited period of time. Moreover, most of the participants have extensive experience in teaching, which may have contributed to the results. As Hattie (2003) reported, expert teachers are more capable of seeking and giving feedback, and also monitoring their students’ learning than novice teachers. Conducting studies on novice teachers would help identify their specific needs for training on instructional variables, and design school-based interventions to meet these professionals’ needs.

Elementary and middle school teachers’ conceptions of homework feedback were mapped, but the role of students in the homework feedback process should be further researched. Further investigation may want to explore elementary and middle school students’ conceptions of homework feedback and compare their responses with current findings. The information provided would be useful to learn how students understand (e.g., in what ways students perceive teachers’ homework feedback practices as helpful, see Xu, 2016) and cope with the homework feedback given in class. Examining the (mis)alignment of both conceptions of homework feedback (elementary and middle school teachers and students) may help deepen the understanding of the impact of homework feedback and further examine the differential relationship between doing homework and academic achievement at these two school levels (see Cooper et al., 2006). The results, although promising, should be further investigated in different school grades and subjects. At this level, however, they may be useful to researchers looking for an in-depth understanding of homework feedback and willing to explore new research topics on the “last but not least” aspect of the homework process.

## Ethics Statement

This study was reviewed and approved by the ethics committee of the University of Minho. All research participants provided written informed consent in accordance with the Declaration of Helsinki.

## Author Contributions

JC and PR substantially contributed to the conception and the design of the work. JC was responsible for the literature search. JC, AN, TM, JN, and TN were responsible for the acquisition, analysis, and interpretation of data for the work. PR was also in charge of technical guidance. JN made important intellectual contribution in manuscript revision. JC wrote the manuscript with valuable inputs from the remaining authors. All authors agreed for all aspects of the work and approved the version to be published.

## Conflict of Interest Statement

The authors declare that the research was conducted in the absence of any commercial or financial relationships that could be construed as a potential conflict of interest.
